# Superior Mesenteric Artery Syndrome Complicated by Nutcracker Syndrome: Diagnostic and Therapeutic Challenges

**DOI:** 10.7759/cureus.112104

**Published:** 2026-07-05

**Authors:** Melika Sarkandi, Marisol A Burciaga, Joseph Awa, Katelyn Brown, K. Dean Gubler

**Affiliations:** 1 Emergency Medicine, Rocky Vista University, Parker, USA; 2 Surgery, Rocky Vista University of Osteopathic Medicine, Rocky Vista University, Ivins, USA

**Keywords:** left renal vein compression, nutcracker syndrome, sma syndrome, superior mesenteric artery syndrome, vascular compression syndrome

## Abstract

Vascular compression syndromes result from compression of blood vessels by anatomical structures, impairing arterial or venous flow. Superior mesenteric artery (SMA) syndrome causes duodenal obstruction due to a narrowed aortomesenteric angle and a shortened distance. Nutcracker syndrome (NCS) involves compression of the left renal vein (LRV) between the SMA and aorta. Both conditions are rare, with concurrent cases infrequently reported. *S*MA syndrome has a notably low incidence, while the true prevalence of NCS is not well defined. Overlapping, nonspecific symptoms often delay diagnosis and increase morbidity. We report a 42-year-old woman presenting with two months of worsening abdominal pain, early satiety, nausea, diarrhea, and unintentional weight loss. Physical exam showed left-sided tenderness; laboratory results were unremarkable. Initial imaging, including upper GI series and gastric emptying study, was inconclusive. Symptoms improved with dietary changes but recurred on resuming normal intake, suggesting a structural cause. Computed tomography findings revealed a reduced aortomesenteric angle of 28° and a distance of 3.5 mm with duodenal narrowing, greater than 50% compression of the LRV, dilation of the gonadal veins, and pelvic varices, consistent with concurrent SMA syndrome and NCS. The patient was counseled on conservative management, including nutritional rehabilitation, with surgical options including duodenojejunostomy and renal vein procedures for refractory cases. Close follow-up was planned to monitor response and assess surgical need. This case underscores the importance of clinical suspicion for vascular compression syndromes and the value of advanced imaging when routine tests are inconclusive.

## Introduction

Superior mesenteric artery (SMA) syndrome is a rare cause of duodenal obstruction resulting from extrinsic compression of the third portion of the duodenum between the SMA and the aorta. This compression occurs when the aortomesenteric angle becomes critically narrowed, reducing the aortomesenteric distance and obstructing duodenal transit. Von Rokitansky first described the condition in 1842, and it was later termed Wilkie's syndrome following Wilkie's comprehensive characterization of 75 cases in 1927 [1]. Other names include aorto-mesenteric compass syndrome, arteriomesenteric duodenal obstruction, and cast syndrome, as application of body casts used in conditions such as scoliosis and hip displacement is a recognized cause [1,2]. Patients typically present with postprandial abdominal pain, nausea, vomiting, early satiety, and unintentional weight loss, symptoms that frequently overlap with functional gastrointestinal disorders, contributing to significant diagnostic delay [2,3]. 

Nutcracker syndrome (NCS) is caused by compression of the left renal vein (LRV) between the aorta and the SMA, resulting in impaired blood flow [3]. NCS is divided into anterior and posterior variants, with the anterior type being more prevalent and more frequently observed in women [3]. It typically presents with left flank pain, hematuria, and pelvic congestion [3]. 

The coexistence of these two syndromes is mechanistically linked by their shared anatomical substrate: a narrowed aortomesenteric space. This commonality simultaneously predisposes to duodenal compression in SMA syndrome and LRV compression in NCS, making their concurrent presentation anatomically expected rather than coincidental. Nevertheless, concurrent SMA syndrome and NCS remains exceedingly rare, with fewer than 30 cases documented in the literature to date [3]. Their overlapping and nonspecific symptom profiles create significant diagnostic challenges. We present this case to highlight the importance of maintaining clinical suspicion for concurrent vascular compression syndromes in patients with persistent, unexplained gastrointestinal symptoms and to demonstrate the diagnostic value of advanced cross-sectional imaging when routine workup is inconclusive. 

This case report was previously presented as a conference poster at Rocky Vista University College of Osteopathic Medicine Spring Research Day on May 21st, 2026. 

## Case presentation

A 42-year-old woman presented with an unintentional weight loss of 10 pounds and chronic abdominal pain of two months’ duration, with acute exacerbation lasting 10 days (Table 1). She reported sharp, stabbing epigastric pain and early satiety after small meals. Her symptoms partially improved with adherence to a gluten-free and restricted diet but worsened with nonadherence. She reported nausea, chest heaviness, chills, fever, and diarrhea managed with loperamide.

**Table 1:** Patient demographics, vital signs, physical examination findings, and laboratory results. Reference ranges based on standard institutional values. ALT: Alanine aminotransferase; CBC: complete blood count; CMP: comprehensive metabolic panel; SPO2: peripheral oxygen saturation.

Parameter	Value	Reference Range
Demographics		
Age	42 years	—
Sex	Female	—
Weight	124 lbs (56.2 kg)	—
Physical Examination		
Abdominal tenderness	Left upper and lower quadrants, no guarding	—
Remaining systems	Within normal limits	—
Vital Signs		
Blood pressure	145/55 mmHg	<120/80 mmHg
Heart rate	78 bpm	60–100 bpm
Temperature	36.8°C (98.3°F)	36.1–37.2°C (97°F –99°F)
Respiratory rate	14 breaths/min	12–20 breaths/min
SpO2	97%	≥95%
Laboratory Findings		
CBC	Within normal limits	—
CMP	Within normal limits	—
ALT	Mildly elevated	7–56 U/L
Hepatitis B surface antibody	Reactive	Non-reactive
Remaining hepatitis serologies	Non-reactive	Non-reactive
Gastric Emptying Study		
1 hour	74%	<90%
2 hours	25%	<60%
3 hours	3%	<10%

On physical examination, vital signs were notable for an elevated systolic blood pressure of 145/55 mmHg. All other parameters were within normal limits. The abdomen was tender in the left upper and lower quadrants. The remainder of the exam was unremarkable. Her past medical history was significant for gastroesophageal reflux disease (GERD), inflammatory bowel disease (IBD), and pseudocholinesterase deficiency. 

Diagnostics and assessments**

Laboratory assessment included a normal complete blood count and comprehensive metabolic panel. The patient had a clinically insignificant mild elevation of alanine aminotransferase (ALT); however, the exact numerical value is not available for reporting. Hepatitis serologies revealed a reactive hepatitis B surface antibody consistent with immunity. The rest of the panel was within normal institutional reference ranges. 

Double-contrast upper GI series with small bowel follow-through did not demonstrate definitive duodenal obstruction. A subtle caliber change of the duodenum prompted further evaluation. A gastric emptying study using Tc-99m sulfur colloid labeled egg meal demonstrated normal retention (74% at 1 h, 25% at 2 h, and 3% at 3 h), consistent with normal gastric emptying. 

**Figure 1:**
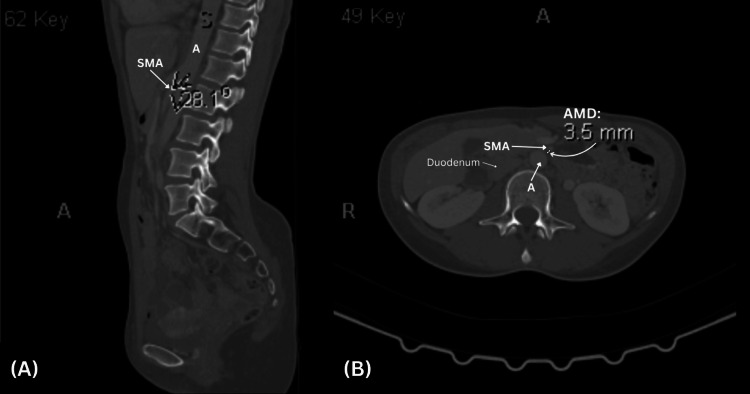
CT abdomen and pelvis with contrast revealing narrowing of the duodenum between the aorta and SMA. (A) Contrast-enhanced CT in sagittal projection demonstrates an aortomesenteric angle of 28° between the superior mesenteric artery (SMA) and the aorta (labeled ‘A’). (B) Contrast-enhanced CT in axial projection demonstrates an aortomesenteric distance (AMD) of 3.5 mm between the SMA and the aorta.

CT abdomen and pelvis with contrast revealed narrowing of the duodenum between the aorta and SMA, with an aortomesenteric distance (AMD) of 3.5 mm and an aortomesenteric angle of 28° without proximal duodenal dilatation (Table 2, Figure 1). Normal aortomesenteric angles are reported to be between 38° and 56° [4], though recent studies suggest ~90° as the true normal value [5]. An angle of 28° (below the lower limit of normal) and AMD of 3.5 mm (below the 8 mm cutoff) support the diagnosis of SMA syndrome [6]. 

**Table 2:** CT measurements and diagnostic thresholds for SMA syndrome and nutcracker syndrome. CT measurements and diagnostic thresholds for superior mesenteric artery (SMA) syndrome and nutcracker syndrome (NCS). Reference thresholds: aortomesenteric angle normal range 38°–56° [4]; aortomesenteric distance (AMD) normal threshold ≥8 mm [6]; beak sign defined as abnormal when present [7]; gonadal vein diameter threshold <5 mm [7], derived from left gonadal vein reference data and applied bilaterally; pelvic varices defined as abnormal when present [7]. LRV: Left renal vein

Measurement	Patient Value	Reference Threshold	Interpretation
Aortomesenteric angle	28°	38°–56° (normal)	Below the lower limit of normal, consistent with SMA syndrome
Aortomesenteric distance (AMD)	3.5 mm	≥8 mm (normal)	Below normal threshold, consistent with SMA syndrome
LRV beak sign	Present	Absent (normal)	Abnormal, consistent with NCS
Left gonadal vein diameter	10 mm	5 mm (normal)	Dilated
Right gonadal vein diameter	9 mm	5 mm (normal)	Dilated
Pelvic varices	Large, bilateral	≤6 mm, non-tortuous (normal)	Abnormal, present bilaterally

**Figure 2:**
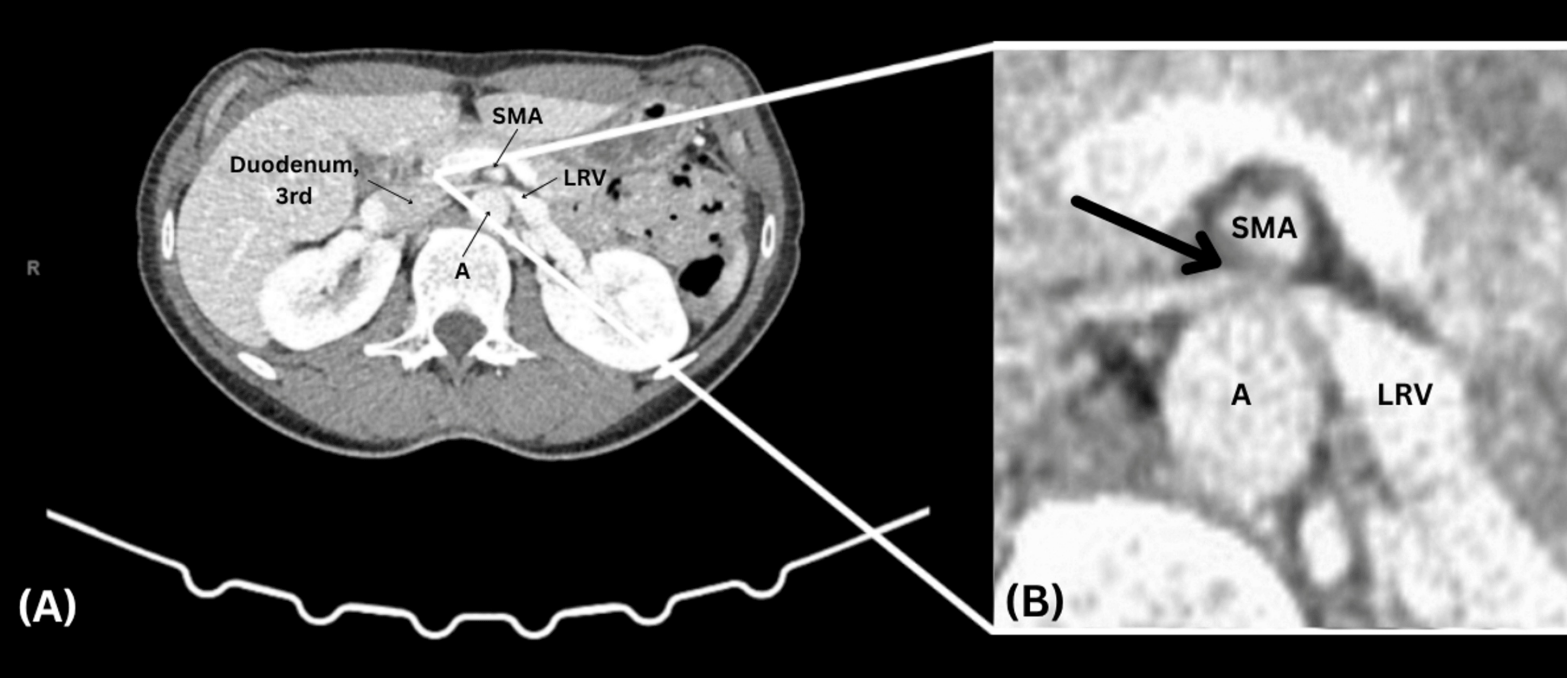
Contrast-enhanced CT demonstrating compression of the duodenum and LRV. Contrast-enhanced CT in axial projection. (A) Compression of the third portion of the duodenum consistent with superior mesenteric artery (SMA) syndrome, with a reduced aortomesenteric angle. (B) Narrowing of the left renal vein (LRV) compressed between the aorta (labeled 'A') and the SMA, with a characteristic beak sign (arrow).

The compressed LRV demonstrated a characteristic “bird-beak” sign, consistent with NCS (Table 2, Figure 2) [7]. The LRV was narrowed >50% between the aorta and SMA, with dilation of the left gonadal vein (10 mm), right gonadal vein (9 mm), and large bilateral pelvic varices [7]. Other abdominal organs were unremarkable. 

Treatment and follow-up

The patient was counseled on conservative and surgical options, including nutritional rehabilitation, weight gain, duodenal bypass, and renal vein reimplantation. Given that her presentation and symptoms did not meet the criteria for a refractory case, conservative management was recommended as the initial approach. The patient did not return for a follow-up. No further outcome data were available at the time of publication.

## Discussion

**Figure 3:**
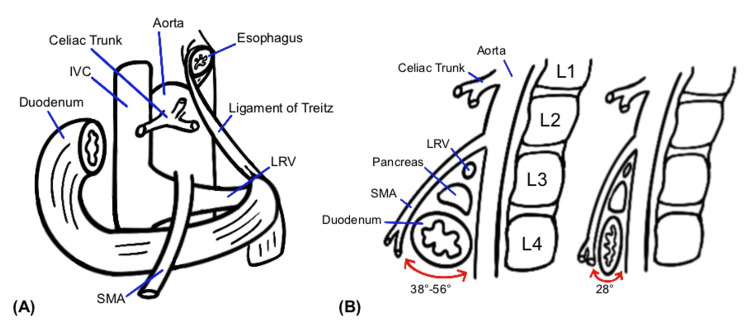
Anatomical illustration of SMA syndrome and NCS. (A) Anatomical illustration of the SMA-aorta relationship with the duodenum and left renal vein (LRV). (B*, Left*) Normal anatomy. (B*, Right*) Duodenal compression consistent with SMA syndrome and LRV compression consistent with nutcracker syndrome. Original illustration hand-drawn by Marisol Burciaga, Rocky Vista University College of Osteopathic Medicine, using Procreate. No AI-assisted tools were used in the creation of this figure. SMA: Superior mesenteric artery; NCS: nutcracker syndrome; IVC: inferior vena cava

SMA syndrome is an uncommon disorder in which the duodenum is compressed between the SMA and the aorta (Figure 3) [1]. Its incidence is estimated at 0.1-0.3%, most commonly affecting adolescents and young adults aged 10 to 39, with familial clustering also reported [8]. Women are more frequently affected (3:2 ratio) [8,9]. Predisposing factors for SMA syndrome include rapid weight loss, gastrointestinal dysmotility, cachexia, visceroptosis, and abdominal wall laxity, all giving rise to narrowing of the aortomesenteric angle [8,10]. In rare cases, proximal duodenal obstruction from SMA syndrome may impair biliary drainage by compressing the distal common bile duct or by causing sphincter of Oddi dysfunction, resulting in cholestasis and pancreatitis [11]. This complication may present as elevated liver enzymes [11]. 

NCS derives from external compression of the LRV between the SMA and aorta, resulting in impaired venous return to the inferior vena cava. While the estimated prevalence is unknown, there have been increased instances in women [3,7]. The coexistence of both syndromes is rare, with only a limited number of cases documented in the literature [3,11]. 

The patient's past medical history was significant for GERD and IBD. Due to the nonspecific nature of her presenting symptoms, including acute abdominal pain, nausea, weight loss, and diarrhea, SMA syndrome is frequently misattributed to conditions such as irritable bowel syndrome, gastroenteritis, or peptic ulcer disease [12,13]. The patient's pre-existing IBD diagnosis may have further complicated the clinical picture, as its hallmark symptoms, including abdominal pain, diarrhea, and nausea, significantly overlap with those of SMA syndrome, potentially contributing to diagnostic delay. Modification of her diet due to such diagnoses may have contributed to malnutrition and unintentional weight loss, which is recognized as a predisposing factor for SMA syndrome [8,13]. Additionally, the patient's history of pseudocholinesterase (butyrylcholinesterase) deficiency warrants close consideration. This deficiency increases sensitivity to neuromuscular blocking agents, specifically succinylcholine and mivacurium, prolonging neuromuscular paralysis and requiring modified anesthetic planning should surgical intervention become necessary [14]. 

Notably, the patient's blood pressure of 145/55 mmHg represented a wide pulse pressure of 90 mmHg, which was documented but not formally investigated during this presentation. While this finding may reflect underlying arterial stiffness or valvular pathology, it was considered incidental in the context of the primary vascular compression diagnosis, and no further workup was pursued. Similarly, while the patient reported subjective fever and chills, her documented temperature at presentation was 36.8°C, consistent with an afebrile state. The reported chills may reflect an inflammatory response associated with duodenal obstruction and mesenteric venous congestion rather than an infectious process. 

Although the upper GI series did not demonstrate definitive duodenal obstruction and CT revealed narrowing without proximal duodenal dilatation, together these findings reflect the partial and positional nature of duodenal compression in this case, consistent with an early or mild presentation of SMA syndrome in which complete obstruction may not yet be established. 

Nutritional rehabilitation and positional therapy are the first-line recommended treatments; however, anticoagulation was also considered to reduce the risk of thrombus formation given the hemodynamic consequences of duodenal obstruction in SMA syndrome, including modified flow within the mesenteric vasculature, which carries a significant risk of thrombotic complications [13]. Although anticoagulation was discussed as a precautionary measure to prevent ischemic complications during recovery, it was ultimately not implemented as the patient was lost to follow-up prior to initiation of any formal treatment plan. 

**Figure 4:**
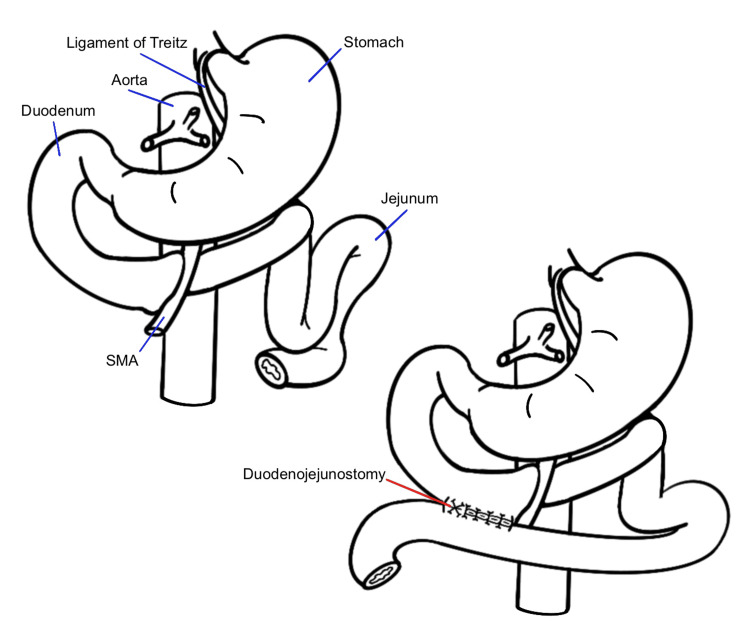
Anatomical illustration of SMA syndrome and duodenojejunostomy. Anatomical illustration of superior mesenteric artery (SMA) syndrome pathophysiology (*top left*), with the duodenum compressed between the SMA and aorta. Duodenojejunostomy procedure (*bottom right*) is the preferred treatment. Original illustration hand-drawn by Marisol Burciaga, Rocky Vista University College of Osteopathic Medicine, using Procreate. No AI-assisted tools were used in the creation of this figure.

For refractory cases, surgical treatment may be considered. Duodenojejunostomy is regarded as the preferred approach, particularly in severe cases (Figure 4) [10,15]. Alternative procedures, including gastrojejunostomy, division of the ligament of Treitz, and vascular reconstruction, are available but are associated with increased risk [15]. 

This case report presents a female patient with the co-occurrence of SMA syndrome and NCS. While only supportive measures were taken, this case emphasizes the importance of timely detection and noninvasive therapies that can help with the management of concurrent vascular compression syndromes. 

Limitations

This case report has several limitations worth acknowledging. Exact numerical laboratory values were not available for inclusion due to limited access to the complete medical record, precluding precise quantitative reporting of individual results. Most importantly, follow-up data were not available, and the treatment course ultimately pursued by the patient remains unknown. While conservative and surgical options were discussed during her care, we are unable to report on patient outcomes. This limits this manuscript's ability to fully illustrate the clinical course of concurrent SMA syndrome and NCS beyond the point of diagnosis. As with any single case report, the findings cannot be generalized to a broader patient population, and the rarity of this concurrent presentation precludes any comparative analysis. Future prospective studies with documented follow-up would be valuable in better characterizing optimal management strategies for patients presenting with both conditions simultaneously. 

## Conclusions

This case illustrates the diagnostic complexity of SMA syndrome when coexisting with NCS. Imaging findings of a reduced aortomesenteric angle and distance, along with LRV compression, highlight the need to consider vascular compression syndromes in patients with persistent, nonspecific gastrointestinal complaints.

Early recognition prevents unnecessary interventions and prolonged morbidity. Conservative management remains the first-line approach, though the optimal treatment course in concurrent presentations warrants further investigation. It should be noted that follow-up data were unavailable for this patient, and the treatment ultimately pursued remains unknown, limiting conclusions that can be drawn regarding management outcomes in this specific case. Reporting rare presentations such as this supports the growing literature and may increase clinician awareness, facilitating earlier diagnosis and individualized treatment strategies. 
